# Women, Mobility, and Education in Twentieth-century England and Wales: A New Analytical Approach

**DOI:** 10.1093/tcbh/hwab037

**Published:** 2022-01-06

**Authors:** Christina de Bellaigue, Eve Worth, Charlotte Bennett, Karin Eli, Stanley Ulijaszek

**Affiliations:** University of Oxford, UK; University of Oxford, UK; University of Auckland, New Zealand; University of Warwick, UK; Institute of Social and Cultural Anthropology, University of Oxford, UK

## Abstract

The twentieth century saw substantial changes in the educational and occupational opportunities available to women in Britain. These may have been supposed to foster new patterns of female mobility. Yet studies of women’s intergenerational mobility are rare and tend not to focus on education. This article develops a historically informed gauge of educational attainment—the Educational Cohort Code (ECC). Applying that gauge to the experiences of women in twentieth-century UK, we make two key claims: first, that despite the prevalence of narratives of progress and mobility in individual and collective accounts of women’s education, there were considerable intergenerational continuities in women’s educational status across the period. Second, that the expansion of educational opportunities across the twentieth century had a differential impact for women and for men and that this differentiation destabilizes categorizations of class solely based on male occupational hierarchies. By applying the ECC method to family data, rather than focusing only on individuals, the article identifies trends within families and the possible influence of family cultures of education and employment on intergenerational mobility.

## Introduction

This article develops a new method of capturing and investigating intergenerational educational mobility, using the analysis of women’s experiences in twentieth-century Britain as a test case. The analysis brings together two new datasets: a historical dataset that captures changes in educational policy as revealed through manpower tables and equivalent information published in the 1971, 1991, and 2001 British censuses, and a socio-epidemiological and anthropometric dataset, with occupational, educational, behavioural, well-being, and family history data collected from ninety participants in the Oxford Biobank.^1^ Drawing on these data, we develop a new way to measure educational attainment based on a gauge that we term the Educational Cohort Code (ECC) that is more historically sensitive than simple participation rates or school-leaving age. This approach permits us to compare educational qualifications across generations in a way that takes account of developments in the educational landscape and in the cultural and social significance of different qualifications over time. The ECC method allows us to shed new light on patterns of female experience in the twentieth century, a period that saw considerable expansion in the educational and occupational opportunities available to women.^2^ It allows us to consider women’s relative upward educational mobility, understood as the likelihood of an individual’s highest educational attainment being higher than that of their mother. By using the ECC, this article reveals that while individual and collective narratives of women’s education might emphasize gains in educational attainment across the century, there were considerable intergenerational continuities in women’s educational status after 1900. Second, it demonstrates that the expansion of educational opportunities across the twentieth century had a differential impact for women and for men and suggests that this differentiation destabilizes categorizations of class solely based on male occupational hierarchies. Finally, by applying the ECC method to family data, rather than focusing only on individuals, the article identifies trends within families and the possible influence of family cultures of education and employment on intergenerational mobility.^3^

Despite the significance of the changes to women’s education and occupation prospects in the twentieth century, most existing sociological and historical analyses have not directly addressed the question of women’s mobility in this period.^4^ This absence is partly a function of the historically contingent development of research into social mobility and social structure which took the adult employed male as norm.^5^ The maleness of the scale of social mobility analyses was exacerbated by the persistence of nineteenth-century ideas about women’s class position being determined by their distance from the labour market, rather than their engagement with it.^6^ Such assumptions later affected the parameters of the key studies of the post-war decades which collected data only on male subjects, contending that female social class could best be defined by a woman’s father’s, and then husband’s, occupation.^7^ This bypassed the need to study women in their own right or to view them as forming their own class/mobility profiles, despite the massive entry of women into the labour market and into secondary and higher education in the post-war period, both of which factors might have been expected to demand attention to women’s social mobility.^8^

The need for research examining women’s mobility is highlighted by the small number of existing studies that do focus on women. This research has pointed to ways in which mobility patterns are gender-specific, highlighting the differences between male and female social and educational mobilities.^9^ It also emphasizes how women contribute to male mobility strategies, and to social class formation. Women might have a significant impact through their sponsorship of their sons’ education, and through marriage in consolidating the class position of their husbands, especially key when those husbands were themselves mobile.^10^ The importance of women as agents of male mobility is thus increasingly recognized, as is the way mobility patterns are gendered.

Significantly, those studies which focus in more detail on women’s mobility in their own right, and move beyond a focus on marital mobility, have also highlighted the importance of education and educational mobility for women both in terms of their occupational trajectory, and in terms of their subjective class identity. Examining women’s mobility in the 1930s, Dyhouse has argued that the implications of higher education were very different for women and for men. She found that men graduates of the 1930s were very frequently upwardly mobile. This, however, was not the case with women graduates in the context of a gendered labour market, though she emphasized the extent to which sending women to university ‘paid off’ in terms of ‘personal and cultural enrichment’.^11^ This divergence reoccurs repeatedly in the existing research on women’s social mobility. There is often a discrepancy between female educational and occupational mobility. Sapsford and Abbott argue women’s post-secondary qualifications are the best guide to how women define their own class. They suggest what matters for women is their *potential* in the labour market.^12^ Nevertheless education retained its value in the eyes of those women graduates in part as facilitating the transmission of advantage to their own children. Similarly, though approaching the question from a different perspective, qualitative sociological research by Steph Lawler, Valerie Walkerdine, and Diane Reay has shed significant light on the gendered experience of mobility for women, and drawn particular attention to the importance of education in both the process and experience of social mobility for women.^13^ More recently, Erzsebet Bukodi’s work has highlighted the greater, and increasing, importance of qualifications for women, than for men, in relation to occupational mobility.^14^ In addition, other research suggests that while occupational class may be an appropriate proxy for social class among men, among women and people of colour, mobility is influenced more by education, and is more relational, with family origins, marriage partnerships, friendships, and the broader community, as key factors.^15^ Education then emerges as a theme of critical importance in both the process and experience of social mobility for women, but one that has been somewhat neglected in quantitative sociological analyses, and has also not been the focus of sustained historical analysis.

Alongside the need for more studies of women’s mobility, we need analyses that are more attentive to the changing meaning and impact of different educational qualifications and experiences over time. Historical studies of the school leaving age in Britain has emphasized in particular how changing legislative contexts in post-war Britain affected the demographics of educational attainment.^16^ Yet sociological studies of educational mobility have tended to use school-leaving age data in ahistorical ways.^17^ Recently Erzsebet Bukodi and John Goldthorpe have developed more nuanced analyses that consider education as a positional good, in the sense that the benefit of particular educational attainments is not absolute but determined by the amount of education an individual attains, relative to others; this has helped to underline the changing value of different qualifications at particular historical moments. Their interest, however, centres on calculating relative rates of qualification, and on the relationship between educational and occupational class (which they measure using occupational classification schemes), and their study does not attend closely to changes in education policy, or to the educational and social value ascribed to different qualifications in different periods.^18^

## The Educational Cohort Code and Intergenerational Comparisons

Existing studies of occupational and educational mobility, then, do not offer sufficiently nuanced ways of identifying trends in intergenerational female social mobility across the twentieth century. At the same time, most studies of intergenerational educational achievement have tended either to compare length of school-life, or the proportion of the population at any given time having secured particular educational qualifications, in ways which do not take account of the changing meaning and value of these different educational experiences and attainments. Thus, comparing the proportion of young women from UK aged sixteen remaining in full-time education in 1951 (19 per cent) with the proportion of young women aged sixteen in UK classified as students in 1981 (75 per cent), suggests that the period saw absolute educational mobility in the sense that a higher proportion of sixteen-year-olds looked to be staying on in secondary education in 1981.^19^ This comparison, however, obscures the difference between the situation in 1948, when the statutory school leaving age was only just being raised from fourteen to fifteen, and 1980, when the statutory school leaving age was sixteen (as it had been since legislation in 1972).^20^ The proportion of the population staying in school to age sixteen did increase substantially in this period, yet this did not necessarily reflect greater equality of opportunity. Rather it reflected a change in the educational landscape which would also have impacted the value and status of that educational experience.^21^ Those who stayed in school until age sixteen in 1948 represented a minority of sixteen-year-olds, who—in the context of the time—were unusually well-educated, with educational experiences qualifying them for a range of skilled and semi-skilled white collar and salaried occupations. Those who left school at age sixteen in 1970—in the context of the 1970s—were relatively lacking in educational experience and qualifications and, in that labour market, were less likely to enter the salariat.^22^ A more nuanced approach to the statistical data on educational qualifications and experiences, which contextualizes them not only in relation to relative scarcity, but also in relation to legislation and to the wider historical context, is needed to capture the standing of different educational attainments across generations. The ECC offers this more nuanced approach, allowing us to start comparing the standing of different educational qualifications across the generations in ways that both account for their relative scarcity and also attend to the specificities of schooling, training, higher education and qualifications, of gender norms, and of the labour market in different historical contexts.

In order to develop the ECC, we drew on a historical dataset that delineates changes in educational policy as captured through manpower tables and equivalent information published in the 1971, 1991, and 2001 British censuses.^23^ This information was then related to a socio-epidemiological and anthropometric dataset of women participants in the Oxford Biobank, born 1956–83. This sample collected together occupational, educational, behavioural, well-being, and family history data from ninety women participants in the Biobank who accepted an invitation to respond to a detailed socio-epidemiological questionnaire.^24^ On the basis of these sources, and of detailed statistical and historical research into the post-war educational landscape, our analysis historicizes the key indicators of education, considering educational attainment and qualifications as positional goods, whose value depended on the wider educational context and labour market, but might also contribute to class identity and intergenerational mobility in less tangible ways. By these means we begin to chart the intergenerational educational mobility of women born across the twentieth century and highlight the more variable and a-linear patterns of women’s mobility.^25^ Unfortunately, though the research of Heidi Mirza, Tracey Reynolds, Suki Reynolds, Linda McDowell, and others highlights the impact of race and ethnicity on women’s classed identities, educational and occupational mobility, and experience, our data does not permit detailed reflection on how raced identities impacted the women in the Biobank sample.^26^ The subjects were almost exclusively white British, and the patterns we identify reflect the experiences of white women without explicitly shedding new light on the ways in which their whiteness shaped their experiences.

**Figure 1:**
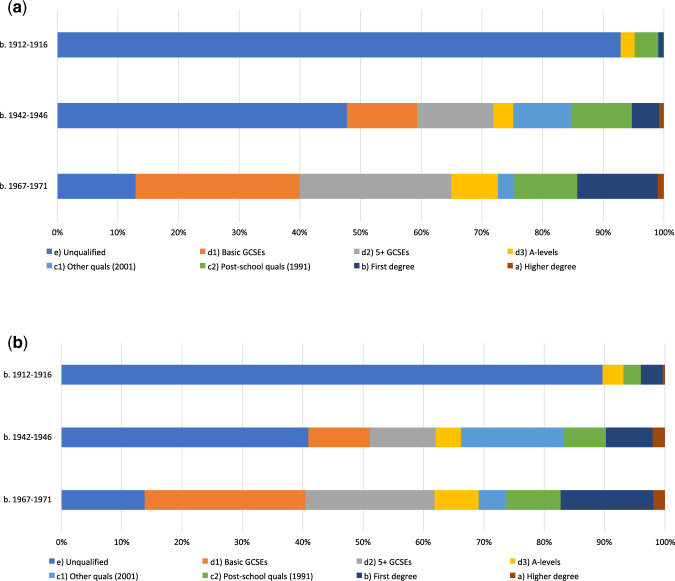
Distribution of highest qualification for the median birth range of each cohort The 1971 manpower surveys identified five categories of qualification which we adopted as the starting point for our classification. It distinguishes between five levels: a (higher university degrees); b (first degrees and equivalent); c (qualifications attained over age eighteen, below degree level); d (holding GCE A-Level or equivalent); and e (persons not qualified). We used the 1971 manpower survey provided information about the highest qualification achieved of women born between 1912 and 1916 (capturing the grandmothers’ generation, who had a median birthdate of 1915). For the mothers’ generation (who had a median birthdate of 1943) we drew on information about those born 1942–46 from the 1971 and 1991 manpower surveys and the 2001 census. This ensured that we were able to capture qualifications achieved later in life. In order to capture the subject’s generation (who had a median birthdate of 1969), we drew on information about those born 1967–71 from the 1991 manpower survey and the 2001 census. The 2001 census employed different categories to the a–e categorization used in the 1971 manpower survey, differentiating between levels 1–4. We thus used a combination that scaled between the 2001 and 1991 data to produce an analysis of the distribution of qualifications that allowed for eight levels of highest educational qualification, differentiating between those with no qualifications and those with ‘Basic’ GCE O-Level/GCSE qualifications or equivalent and those with 5+ O-Level/GCSE qualifications or equivalent. Given the reliance on data from the 2001 Census to perform this operation, which would capture only a very few of the 1912–16 cohort, it was decided to retain the categorizations of the 1971 manpower survey for the grandmothers’ generation. While this is unfortunate in that it leaves hidden any distinction between those of the 1912–16 generation who obtained the School Certificate and those who obtained a Higher School Certificate, since these qualifications were still in their infancy in the interwar period, and only slowly becoming strongly correlated with further educational or employment opportunities, this lack of differentiation in our earliest cohort does not fundamentally undermine the analysis.

To calculate the ECC, we first measured the distribution of British women’s educational achievements across the twentieth century.^27^ Having collected information about the highest qualification achieved in the population as a whole, we examined the distribution of different kinds of educational qualification achieved by age cohorts corresponding to the median birthdates of the Biobank subjects (1969), their mothers (1943) and grandmothers (1915). We were then able to plot the percentage, for each birth cohort, of women and men in the population according to the highest qualification they achieved as seen in Fig. 1.

As Fig. 1a suggests, the distribution of qualifications among the general female population changed significantly over the course of the twentieth century, though those obtaining qualifications beyond A-Level or equivalent have remained a minority of the population as a whole. Figure 1 shows the proportion of women obtaining qualifications growing over the course of the century, and from a lower base than their male counterparts. It also reveals fluctuations in the character of the qualifications obtained. Thus, women in the 1942–46 cohort securing qualifications above A-Level, including vocational qualifications, were a substantial group (20 per cent of women in the age cohort). By the 1980s (when those born between 1967 and 1971 would have been reaching their highest level of qualification), the proportion of women attaining ‘Post-school qualifications’ and ‘Other qualifications’ had dropped to 13 per cent and more women were securing ‘First degree or equivalent’ qualifications (from 5 per cent of women in the maternal cohort up to 13 per cent of women in the subject cohort). This is in line with what has been observed by Carol Dyhouse, and reflects the expansion of Higher Education following the Robbins Report, and the transition to university-based teacher- (and later nursing-) training.^28^ When compared to Fig. 1b which reveals the distribution of qualifications among the male population for the same cohorts, it becomes clear that the expansion in university education for men occurred earlier on; the percentage of men with a first degree or equivalent in the 1942–46 cohort was 8 per cent as compared to 5 per cent of women in the same cohort. Such disparities reflect the importance of accounting for gendered patterns in education and training which will be explored in more detail in Section III below. Figure 1 thus reveals a substantial increase in the proportion of the population securing qualifications over the course of the century. It also suggests, however, that the implications of such qualifications for each generation would have been substantially different. The distribution of qualifications across the cohorts provides a clear sense of the changing educational landscape for women over the course of the twentieth century.

Having characterized the distribution of educational qualifications in the general population for each of our three generations (Fig. 1), our next step was to translate these distributional patterns into a range that would allow us to analyse the attainments of the grandmothers, mothers and subjects in the Biobank data in relation to the general population in their respective cohorts. To that end, we calculated the midpoint of the percentage of women securing each qualification-type in each cohort. This generated a figure that could be used to represent the frequency of different educational experiences within each cohort——an Educational Cohort Code (ECC)—that we could attribute to each individual educational experience identified by the respondents in the Biobank sample, when they answered questions about their own educational qualifications, and those of their mothers and grandmothers.^29^ This process of attributing an ECC to the experiences described by the Biobank subjects reflected our historical understanding of the contemporary educational landscape. Where no information was provided for the mother and/or grandmother we attributed to the figure for whom the information was missing the most common educational level for women of that cohort. In this way, we were able to codify the educational experiences of three generations of women in the ninety families of the Biobank Sample. We were then able to plot the educational trajectory of the three generations of women in a family across the twentieth century, while taking account of the historically specific scarcity value and contemporary prestige of different educational qualifications.

This process was necessarily impressionistic in some cases, particularly with respect to the grandmothers’ cohorts, for whom the information provided was sometimes patchy and lacking in detail. One particular issue was that information about grandmothers was limited to an estimate of their school leaving age, rather than providing details of qualifications obtained. For the 1912–1916 cohort, the minimum school leaving age was fourteen, with those staying on beyond that age able to pursue a growing range of forms of secondary and vocational education and qualifications. It is difficult to develop the fine-grained categorization that distinguishes between those who were unqualified and those who secured qualifications in this early cohort. Given this, and the relative scarcity of secondary education in the population as a whole in the interwar period, it was decided to categorize those leaving school at the age of fourteen as ‘unqualified’ and those identified as leaving age school aged fifteen to eighteen as qualified to ‘A’level or equivalent’. This would necessarily attribute great significance to differences in the school leaving age attributed to their grandmothers by subjects who might have at best, only a hazy knowledge of their relative’s early life.

In some cases, information about mothers was also limited to a sense of the age at which they left school; in these situations, it was decided to categorize those leaving school at age fifteen as ‘unqualified’, given that the qualifications introduced in 1947 were to be taken at age sixteen. Moreover, neither the Biobank nor the Census data allowed for finer-grained distinctions between the multiple different post-sixteen qualifications developed across the period after 1945. First O-Levels were introduced in 1951, replacing the School Certificate. They were primarily intended for grammar school students as the route to the newly introduced academic A-Levels sat at eighteen. During the 1950s some secondary modern schools started to allow their talented students to stay on until sixteen and sit the O-Level exams. Certificates of Secondary Education (CSEs) were introduced in 1965. They were intended to be a more vocational form of qualification to attain at sixteen which the government thought would be better suited to less academic students outside the grammar schools. The difference between these qualifications were both symbolically and materially significant to contemporaries in ways that our data does not capture, though our analysis does nonetheless capture some of the distinctions between different post-war qualifications at sixteen. Both O-Levels and CSEs were replaced by GCSEs in 1986.^30^

A further complication relates to the Teaching Qualifications. For the 1942–46 cohort, such qualifications, obtained in Teacher Training Colleges, would be included under ‘Qualification above A-Level’. For the 1967–71 cohort, given the transition to university-based and post-degree qualifications for teaching, those with a teaching qualification would be categorized under ‘Post-graduate certificate or diploma’. This category-change would thus align with the pattern identified in relation to Fig. 1 above whereby the 1967–71 cohort saw the proportion of women obtaining degree-level qualifications growing and the proportion of those obtaining ‘Qualification above A- level’ declining, but would conceal considerable continuity in terms of the category of education received and the occupational status it would confer.

Finally, the ECC does not account for when qualifications were obtained. As Worth and Thomlinson have noted, it was very common in the post-war period for women to have interrupted educational trajectories, and to return to education later in life, so that their final qualifications might have been achieved long after leaving school.^31^ The ECC cannot capture this dimension of women’s educational experience, though it was clearly significant for the women in the Biobank data set. Forty-seven (52 per cent) of the women in the subjects’ cohort had obtained their highest qualification as mature students.

**Figure 2:**
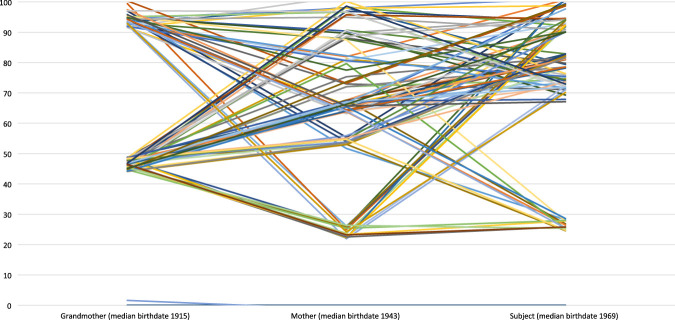
Intergenerational comparison using the ECC of the highest qualification obtained by grandmothers, mothers and subjects in the families of ninety women In order to make patterns in the data more clearly visible in this graph, a uniform random value between –0.5 and 0.5 was added to each point. The effect of this is to prevent identical lines obscuring each other.

Despite these limitations, as Fig. 2 reveals, using the ECC to compare educational qualifications across generations in a way that reflects the relative scarcity of different qualifications reveals a more complex picture than data on the distribution of qualifications would suggest. A direct comparison of the qualifications obtained by women across three generations in most families would suggest a simple upward curve in educational attainment across the generation. Figure 2, which reflects the positional value of different educational qualifications using the ECC, reveals that in fact there was much less clarity in the direction of travel than this comparison would suggest. Instead, there was considerable intergenerational stability in the levels of education secured by the women in most families across the twentieth century. In other words, while there may have been considerable absolute upward educational mobility across the twentieth century (as demonstrated by Fig. 1), Fig. 2 demonstrates that there was significant relative intergenerational stability in terms of educational attainment for a considerable proportion of the Biobank population.

Figure 2 highlights a pattern of relative stability of attainment levels—and the reproduction of educational advantage—among the most educated. Similar continuities are visible among the less educated. It calls into question the idea that educational expansion facilitated simple and direct upward intergenerational mobility. Focusing on case-studies of three families in the Biobank sample further underlines intergenerational continuities in education. By returning to the Biobank data and considering family trajectories, we are also able to explore these patterns in more depth and also to point to the potential influence of internal family traditions underpinning particular intergenerational trajectories.

Table 1 reveals considerable continuity in the educational qualifications of the women in the family of Subject 110, with all three generations of women securing a higher-level ECC, although their educational experiences were very different. All three women were relatively more educated with respect to their peers. As the table also reveals, in the case of the subject and her mother, these women went into occupations defined as professional or semi-professional that educational qualifications provided access to. After the First World War, employment opportunities for women in Britain increased owing to the expansion of light manufacture and of non-manual positions in the service sector.^32^ In the 1930s, becoming a shop assistant, as the subject’s maternal grandmother did, would also have required a higher level of education, and material resources, than factory work or domestic service.^33^ In the next generation, women with a higher level of education might have benefited from the lifting of the public sector marriage bar, which began in the 1940s, and from the post-war welfare state expansion which increased the number of lower professional and administrative roles available to women, with part time work in the welfare semi-professions being introduced during further state expansion in the 1960s and 1970s.^34^ White-collar and semi-professional employment opportunities for more educated women clearly expanded during the course of the period, and the women in the family of Subject 110 appear to have pursued such opportunities and the education needed to access them. The limited information available about their men of the family suggests a rough equivalence in educational qualification and occupational status between the grandmother and her spouse, and the mother and her spouse.^35^ The family of Subject 110 highlights the intergenerational stability of the educational attainment of the women in the three generations of this family, and what looks like a tradition of investment in female education that was responsive to the changing labour market.

**Table 1:** Case study of educational continuities in the families of the more educated Biobank subjects

*Biobank110*	Grandmother (maternal)	Mother	Subject
Date of Birth	b.1917	b.1943	b.1975
Qualification	ECC 94.04 Left school at sixteen	ECC 89.74 Technical or professional certificate	ECC 99.51 Postgraduate degree
Occupation	Shop worker	Special Needs Teaching Assistant	Legal executive (currently not formally employed, stay-at-home parent)
Partner information	b.1917 ECC 91.41 Left school at 16 Disabled, unable to work	b.1945 ECC 88.47 Technical or Professional Certificate Insurance consultant	Operations manager £90,000 (household income)

The family of Subject 915, represented in Table 2, provides an example of a similar level of intergenerational continuity at the less educated end of the scale and again highlights the value of the ECC in demonstrating continuity across generations. Both women in the first two generations left school with no qualifications; however, although the subject left school in the mid-1970s with GCSE/CSE/O-Level qualifications and was thus ostensibly more qualified than her mother and grandmother, she remained in the same ECC band as her predecessors. As with the family of Subject 110, the women in this family tended to marry men of roughly equivalent educational and occupational standing.

**Table 2:** Case study of educational continuities in the families of the less educated Biobank subjects

*Biobank915*	Grandmother (maternal)	Mother	Subject
Date of Birth	b.1905	b.1934	b.1961
Qualification	ECC 46.45 Left school at fourteen	ECC 23.88 Did not complete GCSE/CSE/O-Level	ECC 26.46 Completed GCSE/CSE/O-Level
Occupation	Housewife	Cook	Local government officer Household income—£42,000
Partner information	b.1900 ECC 44.84 Left school at 14 Bus driver	b.1930 ECC 24.48 Left school at 15 Mechanical electrician	No information No information Retired

As Fig. 2 demonstrates, using the ECC—a measure of educational attainment as a positional good that is historically contingent—challenges a simple narrative of the expansion of women’s educational opportunities and upward educational mobility across the twentieth century. Tables 1–2, looking in more detail at individual families within the data, reinforce this point, highlighting the continuities in educational attainment over several generations at either end of the spectrum. Despite the prevalence of narratives of progress and mobility in individual and collective accounts of women’s education, there were considerable intergenerational continuities in women’s educational attainments across the period.

Detailed analysis of other cases of intergenerational educational stability in the sample also reveals interesting continuities in the type of education pursued by women, with frequent evidence of a matrilineal tradition. In the family of Biobank Subject 727, for example, both mother and daughter left school pre-A-Level to pursue technical training, with both then operating as self-employed hair-dressers, though the subject had secured GCSEs/O-Level equivalents, whereas the mother had not. In the family of Biobank Subject 769, mother, grandmother and subject all had relatively high ECCs, and in the second and third generations, had pursued vocational training to work, respectively, as a secretary and property-manager, suggestive of a tradition of female education leading to clerical/service occupations.

To the extent that the twentieth century did see increased relative and long-range upward educational mobility for women, analysing ECC trajectories suggests that this was concentrated in the post-war generation—the first to benefit from compulsory, free secondary education. Indeed, the 1944 Education Act was one of the most significant pieces of legislation passed in twentieth-century Britain.^36^ It expanded secondary education and made this stage of education compulsory. This was particularly influential for working-class girls because they were more likely to lose out on secondary education when places were limited and families had to find the money for uniforms and small fees. Thus, of the total of fifty Biobank subjects who obtained undergraduate or further degrees (56 per cent of the total sample of 90), twenty-four (48 per cent of those obtaining undergraduate or further degrees, and 26 per cent of all subjects) had a higher ECC than their mothers. These twenty-four women would thus appear to have benefited from the expansion of secondary, higher and further education opportunities available to women in the post-war period and been upwardly educationally mobile. Significantly, of these twenty-four women, sixteen had secured their highest educational qualifications as mature students, thus confirming the prevalence of interrupted educational trajectories among women in the post-war generations and the significance of opportunities for mature study.^37^

Examining in more detail the situation of some of the subjects whose ECC was significantly higher than that of their mothers confirms the impact of the post-war context of expanding educational opportunity and of an expanding welfare state. It uncovers the frequent pattern of returning to education in adult life. Table 3 details the intergenerational trajectory of Biobank Subject 522, born in 1975, who secured undergraduate and postgraduate nursing qualifications on her return to education. Subject 522 clearly benefited not only from the expansion of higher education (and the recategorization of nursing as a degree subject), and from the opportunity for mature study, but also from the employment opportunities offered by the NHS.^38^ Her mother, born in 1952, had a lower ECC, leaving school having secured O-Levels (thereby securing more qualifications than the grandmother who had left school at age fourteen) but also benefited from the expansion of the welfare state, finding employment as a school administrator.

**Table 3:** Case study of long-range upward educational mobility in the families of Biobank subjects

*Biobank522*	Grandmother (maternal)	Mother	Subject
Date of Birth	b.1919	b.1952	b.1975
Qualification	ECC 46.45 Left school at fourteen	ECC 53.56 Completed GCSE/CSE/O-Level	ECC 99.51 Postgraduate Qualification
Occupation	Unknown	School Administrator	Nursing Practitioner Modern professional £24,000 Undertook UG & PG qualifications as an adult
Partner information	b.1898 ECC 44.84 Unqualified Unknown	b.1948 ECC 64.14 A-Level/equivalent Printing company director	IT Engineer No information £40,000

**Table 4:** Case study of downward educational mobility through marriage

*Biobank275*	Grandmother (maternal)	Mother	Subject
Date of Birth	b.1920	b.1946	b.1970
Qualification	ECC 94.04 A-Level/equivalent	ECC 73.55 A-Level/equivalent	ECC 99.51 Postgraduate Qualification (in progress)
Occupation	Occupation unknown	Housewife	Self-employed Childminder £15,500 Defined as ‘clerical or intermediate’ & part-time studies.
Partner information	b.1917 ECC 91.41 A-Level/equivalent Chartered Secretary	b.1938 ECC 53.56 Completed GCSE/CSE/O-Level Publisher	Communications Director £37,500

**Table 5:** Case study of the importance of qualifications for women in the interrupted career

*Biobank22*	Grandmother (maternal)	Mother	Subject
Date of Birth	b.1930	not recorded	b.1980
Qualification	ECC 94.04 A-Level/equivalent	ECC 99.61 Postgraduate degree	ECC 80.55 Vocational certificate
Occupation	Housewife	Community Learning Coordinator	Procurement Officer £35,000 Occupation 10 years ago: Customer service
Partner information	b.1930 ECC 91.41 A-Level/equivalent Railway Worker	b.1938 ECC 94.1 Undergraduate degree Software developer	Construction manager £35,000

Together, the overall comparison visible in Fig. 2 and the case studies of women in the Biobank sample using the ECC reveals both the classed intergenerational stability of educational attainment across the twentieth century for the bulk of the women in the sample, and the significance of structural factors—the expansion of secondary education and of the welfare state—in facilitating a degree of upward educational and occupational mobility for a significant minority of women in the post-war period. Figure 2 also points to a cluster of families in the Biobank sample who experienced not upward, but downward mobility, a phenomenon that will be further explored below. What Fig. 2 does not suggest, as noted above, is a clear picture of educational expansion producing upward educational mobility across the century.

## Gendered Differences in Educational Attainment

The comparison over three generations of highest qualification achieved by women in these families draws attention to the significance of the movement around mid-century, when the subjects’ mothers were obtaining their qualifications, a point at which, as Fig. 1 revealed, the distribution of male and female educational attainment varied. In order to explore this moment further, we focused on the gendered dimension of educational attainment in the parental and subject generations.

**Figure 3:**
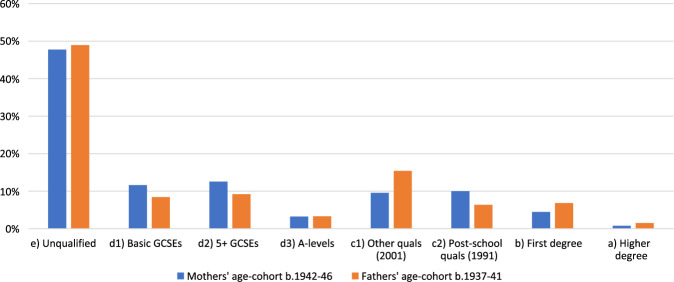
Highest qualification achieved by men and women in the maternal and paternal age cohorts

Our first step was to compare the distribution of qualifications in the population as a whole among men and women born in the median range of the maternal cohort (b. 1942–46) and paternal cohort (b. 1937–41——the median age of the fathers of the subject cohort was slightly older than that of mothers).^39^ As Fig. 3 reveals, there were important differences in the distributions type and level of qualifications achieved by men and women in the two parental cohorts. These patterns were also repeated in a comparison between women in the maternal cohort, and men in the same birth cohort of 1942–46. Women born in the maternal cohort of 1942–46 seem to have been more likely than men born in the paternal cohort of 1937–41 to secure school-based qualifications such as General Certificate of Education (GCE) O-Levels or equivalent, likely benefitting from the expansion of secondary schooling and the extension of school life subsequent to the Butler Act. They were also more likely to obtain the qualifications categorized by the 1991 census ‘qualifications that are: (i) generally obtained at age eighteen and over; (ii) above GCE A-Level standard; and (iii) below UK first degree standard’ and which included teaching and nursing qualifications. Men born between 1937 and 1941, were more likely than women in the maternal cohort to obtain a university degree, but were also more likely to secure qualifications characterized in the 2001 Census data as ‘Other qualifications/level unknown: Other qualifications (e.g. City and Guilds, RSA/OCR, BTEC/Edexcel), Other Professional Qualifications’, and which, in the post-war period, would have included apprenticeships and other vocational training. These findings chime with those of the Cambridge Secondary Education and Social Change Project which found that in the post-war period, boys were more likely than girls to pursue apprenticeships, and that girls pursuing Further Education in the same period would be more likely to enter into teacher training or secretarial courses.^40^

**Figure 4:**
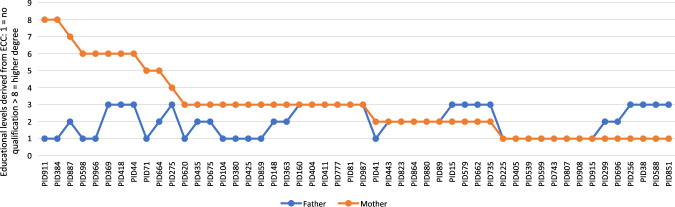
Comparison of the ECC of less educated men and their spouses in the parental cohorts

Our next step was to explore how far the gender differences in educational experience evident in the population as a whole were apparent within particular families. In order to do this, we carried out the same processes we had already adopted to calculate the ECC with respect to mothers in the Biobank sample in order to characterize the educational experiences of fathers in the sample. This allowed us to compare the experiences of wives and husbands (the Biobank subjects’ mothers and fathers) at the mid of the century.

As might be expected, given the findings revealed in Fig. 3, the comparison identified six husbands with university degrees whose wives had not attended university. More surprisingly, it also uncovered a significant proportion of women who were more qualified than their spouses (Fig. 4). Of the fifty women married to husbands with no qualifications or with pre-A-Level qualifications only, twenty-one (42 per cent) were more qualified than their husbands. These figures again serve to underline the importance for women of the expansion of secondary and further education at mid-century.

While women were benefiting from new educational opportunities, as the work of Carol Dyhouse and Helen McCarthy has shown, there was concern in the post-war period about the ‘wastage’ of women’s education. Objections were raised to the situation whereby women received an education of which they could then not realize the potential in the labour market, partly because of the emphasis on domestic femininity and the need to give up paid work on marriage (or, increasingly in this period, when women had young children), and partly because of gendered discrimination in the workplace.^41^ Hitherto, discussion of women’s social mobility has principally focused on marital mobility, suggesting that it was principally through ‘marrying up’ that women might achieve upward social mobility, rather than through their own efforts.^42^ Comparing the Educational Cohort Codes of fathers and mothers in the Biobank Sample suggests that in twenty-four couples (27 per cent of the whole sample), the educational attainment of wives was not matched by their husbands. One interpretation of this would suggest that—in educational terms at least—marriage might signal downward, rather than upward, mobility.

The case of Biobank Subject 275 (Table 4) provides an illuminating example of this pattern. The women in all three generations secured a fairly high level of qualification. In the parental generation, however, the mother, who became a housewife and did not make further use of her qualifications in economic activity outside the home, had a higher ECC than her husband, who worked in publishing. On the face of it, investment in the education of the mother of Biobank Subject 275 was ‘wasted’ both in terms of her position in the labour market and in terms of her marital mobility.

As the work of many scholars suggests, however, this situation was not necessarily interpreted as one of social decline by the women themselves.^43^ Many defended the intrinsic value of the education they had pursued, and their class identity rested significantly on their educational attainments. Steph Lawler has argued that upwardly women often emphasize knowledge, education, taste and lifestyle over occupation in defining their own class position.^44^ Moreover, educational qualifications were often understood as better enabling women to support the educational and occupational aspirations of their husbands and children.^45^ In the case of Biobank Subject 275 it could be speculated that the mother’s educational qualifications facilitated the pursuit of further education by her daughter. The same patterns are apparent in the trajectories of other families in the sample where the mother had achieved a higher level of education than her husband.^46^ The educational attainments of the next generation would echo those of the mothers, and often translated into professional or semi-professional careers, rather than those of the fathers, who tended to work either in the trades, in factories, or in business activities. Of the twenty-four mothers who had a higher ECC than their husbands, sixteen (67 per cent) had daughters who then went on to attain a higher ECC than either their mother or their father. Thirteen of these mothers (54 per cent) worked either in clerical/secretarial occupations, or in education, the NHS, or local government, as did eighteen (75 per cent) of their daughters. These figures suggest the significance for women of educational qualifications and employment in the expanding public sector in the post-war period, the potential influence of mothers on their daughters’ trajectories, and highlight differences between male and female patterns of education and occupation.

This emphasis on educational qualification and training in the lives of women in the Oxford Biobank sample, including for those married to less qualified men, also supports the claim that formal qualifications may be more critical to women’s professional development than to men’s.^47^ Evidence from the subject generation in the Biobank sample suggests that this is further exaggerated by the interrupted nature of women’s educational and occupational trajectories and gendered differences in the kinds of qualification and occupation available to them. Subject 275, for example, who had previously held a higher managerial position, had young children and had shifted to flexible work as a childminder alongside part-time study in pursuit of a post-graduate qualification. It is plausible to suggest that this qualification would eventually facilitate her re-entry to full economic activity outside the home. Similarly, Biobank Subject 22 (Table 5) had pursued a vocational qualification as a mature learner in order to transition to working as a Procurement Officer.

The Biobank data does not allow us to compare directly the educational experiences of husbands and wives in the subject’s generation, but it is possible to compare those of male and female Biobank subjects. It is clear that mature learning was also a significant feature in the lives of male subjects (68 per cent of the forty men in the male sample had returned to education, compared to 52 per cent of the ninety women in the female sample). However, there were significant differences in the ways in which these men and women pursued their learning and in the kinds of qualification they sought. Women mature learners tended to pursue the kind of vocational and professional qualifications pursued by Biobank Subjects 275 and 22, which, as we have seen, might lead to roles in the expanding welfare state and service sector. The twenty-seven women (57 per cent of female mature learners) who did so were in healthcare, legal/clerical services, educational administration, alternative therapies, accountancy, childcare, and library work, having been in lower-level positions in similar or related fields 10 years earlier. Women were also more likely to pursue undergraduate and higher education. The eleven (23 per cent of female mature learners) who did so were in education, healthcare, childcare, and IT. In contrast, men were more likely to receive on the job training: the seven men (26 per cent of male mature learners) who did so were in the police service and the building trades, having been in the same field 10 years previously. The pattern seems to emerge that while mature learning was important for both men and women in the subject generation, more women pursued new vocational qualifications beyond the workplace, whereas more men pursued on the job qualifications. This suggests that while mature learning might secure a route into employment or re-employment for women, for men it was more likely to secure promotion within their existing occupation. We see here an echo of the differences in qualification types pursued by the parental generation, when boys were more likely to pursue apprenticeships. Across both cohorts then, our data highlight gendered differences in the meaning and value of educational qualifications and suggests the ways that educational attainment might inflect class and occupational identity and mobility differently for men and for women.

## Conclusions

Drawing together two datasets and developing a new way to account for women’s educational attainments and mobility, this article has suggested that the twentieth-century expansion of educational opportunity for women had more complex effects than a simple comparison of qualifications over time might suggest. Developing a measure and analysis of education as a historically specific positional good for women highlights the intergenerational stability of educational attainment, the preservation over generations of educational advantage and disadvantage, and of gendered patterns of qualification, despite the extension of schooling and further education, even though we should recognize that such stability in educational attainment might conceal considerably different life experiences. To the extent that relative increased educational mobility can be discerned, our analysis further underlines the significance of the mid-century reforms and the extension of the welfare state in providing both educational opportunity and demand for qualified women to staff this expansion. For the mothers’ generation, this heightened the importance of schooling and formal qualifications which did not necessarily translate directly into labour-market value or into upward marital mobility, but which nonetheless were of considerable significance to them. Exploring family histories within this data seems to point to the prevalence of intergenerational family traditions in education and employment which might also not be otherwise apparent.

Our analysis provides a quantitative dimension to the evidence from interviews of the significance of education and educational mobility to women’s class identity and opportunities in post-war Britain. At the same time, while Fig. 1 suggests that by the 1970s, gender differences in access to higher education and other qualifications were less pronounced, further analysis of patterns of mature learning and the trajectories of women in the Biobank sample suggest that this apparent alignment continued to conceal significant disparities in the educational itineraries of men and women, in the kinds of qualification they secured, and in the meanings of those qualifications. Accounting for women’s educational attainments and mobility in a way that reflects the educational and occupational context in which they were secured, both highlights the intergenerational stability of educational attainment across the century, and destabilizes traditional categorizations of class based on male occupational hierarchies.

## Footnotes

1 The Oxford Biobank is a collection of bio-data from approximately 8,000 healthy men and women living in Oxfordshire, randomly selected for participation through NHS number. It is incorporated into the National National Institute of Health Research Bioresource, and its biological research infrastructure is provided through ten-bed clinical research unit at the Oxford Centre for Diabetes, Endocrinology and Metabolism (OCDEM), which is supported by the NIHR Oxford Biomedical Research Centre. Among other measures, the Oxford Biobank collects anthropometric data, including height, weight, and fat mass distribution measures (collected through DXA scanning). F. Karpe, S. K. Vasan, S. M. Humphreys, J. Miller, J. Cheeseman, A. L. Dennis, and M. J. Neville. ‘Cohort Profile: The Oxford Biobank’, *International* *Journal of* *Epidemiology*, 47 (2018), 21.

2 C. Dyhouse, ‘Education’, in I. Zweiniger-Bargielowska ed., *Women in Twentieth-Century Britain* (London, 2001);.C. Dyhouse, *No Distinction of Sex? Women in British Universities, 1870–**1939* (London, 1995). A. Oram, *Women Teachers and Feminist Politics*, 1900–1939 (London, 1996). F. Hunt, ed. *Lessons for Life: The Schooling of Girls and Women 1850–1950* (1988). S. Spencer, *Gender, Work and Education in Britain in the 1950s* (Basingstoke, 2005); E. Edwards, *Women in teaching training colleges, 1900-1960. A culture of femininity* (London, 2000).

3 Carol Dyhouse’s work provides a rare example of research analysing social mobility which explores family histories. C. Dyhouse, ‘Graduates, Mothers and Graduate Mothers: Family Investment in Higher Education in Twentieth-Century England’, *Gender and History*, 14 (2002), 325–6. C. Dyhouse, ‘Family Patterns of Social Mobility through Higher Education in England in the 1930s’, *Journal of Social History*, 34 (2001), 817–42. See also C. de Bellaigue, ‘Great Expectations? Childhood, Family and Middle-class Social Mobility in Nineteenth-century England’, *Cultural and Social History*, 16 (2019), 29–46.

4 For discussion of this see C. de Bellaigue, H. Mills and E. Worth, ‘Introduction’ and Mike Savage and Magne Flemen, ‘Life Narratives and Personal Identity: The End of Linear Social Mobility?’, *Cultural and Social History*, 16 (2019), 1–11 and 85–101.

5 C. Renwick, ‘Eugenics, Population Research and Social Mobility Studies in Early and Mid-Twentieth-Century Britain’, *Historical Journal*, 59 (2016), 845–67.

6 L. Davidoff and C. Hall, *Family Fortunes: Men and Women of the English Middle Class, 1780-1850* (London, 1987), 273; P. Summerfield, *Reconstructing Women’s Wartime Lives* (Manchester, 1998) 199–249. H. McCarthy, ‘Women, Marriage and Paid Work in Post-war Britain’, *Women’s History Review,* 26 (2017), 46-61. H. McCarthy, ‘Social Science and Married Women’s Employment in Post-war Britain’, *Past and Present*, 233 (2016) 275–6.

7 D. Glass, ed., *Social Mobility in Britain* (London, 1954). J. Goldthorpe, C. Llewellyn, and C. Payne, *Social Mobility and Class Structure in Modern Brita*in (Oxford, 1980) 17–29.

8 P. Payne and G. Abbott, eds, *The Social Mobility of Women: Beyond Male Mobility Models* (London, 1990) is a key exception.

9 Payne and Abbott, eds, *Social Mobility of Women*. S. Dex, *Women’s Occupational Mobility: A Lifetime Perspective* (London, 1987). P. Abbott and R. Sapsford, *Women and Social Class* (London, 1987). D. Gittins, *Fair Sex: Family Size and Social Structure, 1900-32* (London, 1982).

10 D. Bertaux and I. Bertaux-Wiame, ‘Heritage and its Lineage: A Case History of Transmission and Social Mobility over Five Generations’, in D. Bertaux and P. Thompson, eds, *Pathways to Social* *Class: A Qualitative Approach to Social Mobility* (Oxford, 1997) 62–97. P. Thompson, ‘Women, Men, and Transgenerational Family Influences in Social Mobility’, in Bertaux and Thompson, eds, *Pathways to Social Class*, 32–61. L. Davidoff, ‘The Role of Gender in the ‘first industrial nation’ Agriculture in England, 1780-1850’ in R. Crompton and M. Mann, eds, *Gender and Stratification* (London, 1986), 190–214.

11 Dyhouse, ‘Graduates, Mothers’; Dyhouse, ‘Family patterns’.

12 Sapsford and Abbott, *Women*, 75.

13 S. Lawler, ‘Getting Out and getting Away’: Women's Narratives of Class Mobility’, *Feminist Review*, 63 (1999), 3–24. V. Walkerdine, ‘Reclassifying Upward Mobility: Femininity and the Neo-liberal Subject’, *Gender and Education*, 15 (2013), 237–48. D. Reay, ‘Rethinking Social Class: Qualitative Perspectives on Class and Gender’, *Sociology*, 23 (1998), 259–75.

14 E. Bukodi, ‘Education, First Occupation and Later Occupational Attainment: Cross-cohort Changes Among Men and Women in Britain’, *CLS Cohort Studies Working Paper 2009/4* (2009), 20.

15 E. Higginbotham and L. Weber, ‘Moving Up with Kin and Community: Upward Social Mobility for Black and White Women’, *Gender and Society*, 6 (1992), 416–40. In addition, pilot research examining the impact of upward mobility on body weight and health has suggested that gender is key to associations between social mobility and obesity for women in ways that do not relate directly to occupational mobility, and which underscores the need for an approach to social mobility research which takes into account mediators between class and health other than those based on occupation. See K. Eli, F. Karpe and S. Ulijaszek, ‘Using a New Socioepidemiological Questionnaire to Analyse Associations between Intergenerational Upward Social Mobility and Body Fat Distribution: A Pilot Study with the Oxford Biobank cohort’, *Journal of Epidemiolgy and Community Health*, 74 (2020), 981–7.

16 T. Woodin, G. McCulloch, and S. Cowan, *Secondary Education and the Raising of the School Leaving Age* (New York, 2013); Secondary Education and Social Change Project (SESC), University of Cambridge, ‘ROSLA ’ SESC Briefing paper: ROSLA’, October 2017, <https://sesc.hist.cam.ac.uk/wp-content/uploads/2018/01/Briefing-paper-ROSLA.pdf> (last accessed 24.11.2021). P. Mandler, *The Crisis of the Meritocracy: Britain’s Transition to Mass Education since the Second World War* (Oxford, 2020), chapter 6.

17 See L.-A. Vallet, ‘Change in Intergenerational Class Mobility in France from the 1970s to the 1990s and its Explanation’, in R. Breen, ed., *Social Mobility in Europe* (Oxford, 2004) for an example of an analysis using school-leaving age.

18 Y. Shavit and H. Park, ‘Education as a Positional Good’, *Research in Social Stratification and Mobility*, 43 (2016), 1–3. E. Bukodi and J. Goldthorpe, ‘Educational Attainment——Relative or Absolute——as a Mediator of Intergenerational Class Mobility in Britain’, *Research in Social Stratification and Mobility*, 43 (2016), 5–15.

19 Table 54, Percentage of persons in Full-time Attendance at an educational establishment, in *1951 Census: General Report* (London, 1958); ‘Table 2: Usually resident (economic activity) population: economic position and employment status by age by sex (and married women)’ in *Census 1981: Economic Activity* (London, 1984).

20 SESC, ‘ ROSLA’.

21 Mandler, *Crisis of the Meritocracy*, provides the fullest account of the changes in education at school, rather than policy level for the period after 1945.

22 P. Mandler, ‘Educating the Nation: III Social mobility’, *Transactions of the Royal Historical Society*, 26 (2016), 13.

23 Table 1, Population 18–69 years of age by educational attainment, sex, age and area of usual residence, in *Census 1971 Great Britain: Qualified Manpower Tables (10% Sample)* (London, 1976), 1–8; Table 1: Age and sex—Residents aged 18 and over: sex by level of highest qualification and percentage qualified, by age, in *1991 Census: Qualified Manpower—Great Britain* (London, 1994),19–28; The 2001 census data was taken from Nomis on 2 May 2018: ST105—Sex and age by highest level of qualification, England and Wales.

24 The largest random population-based epidemiological cohort in the UK, the Oxford Biobank carries data on health markers, genetics, body composition, social and demographic factors on over 8,000 adults. Eli, Karpe and Ulijaszek, ‘Using a New Socioepidemiological Questionnaire’.

25 Eli, Karpe and Ulijaszek, ‘Using a New Socioepidemiological Questionnaire’.

26 H. Mirza, *Young, Female and Black* (London, 1992). T. Reynolds, ‘Black Women and Social Class Identities’ in S. Munt (ed.), *Cultural Studies and the Working Class* (London, 2000), 82–96. S. Ali, *Mixed-Race, Post-Race. Gender, New Ethnicities and Cultural Practices* (Oxford, 2003), 174–6. L. McDowell, *Working Lives: Gender, Migration and Employment in Britain, 1945-2007* (Oxford, 2013).

27 The following tables were used to generate this data: Table 1: Population 18–69 years of age by educational attainment, sex, age and area of usual residence, in *Census 1971 Great Britain: Qualified Manpower Tables (10% Sample)*, 1–8; Table 1: Age and sex——Residents aged 18 and over: sex by level of highest qualification and percentage qualified, by age, in *1991 Census: Qualified Manpower*—*—Great Britain*,19–28; The 2001 census data was taken from Nomis on 2 May 2018: ST105——Sex and age by highest level of qualification, UK.

28 Dyhouse, *Students*, 98. P. Summerfield, ‘Women in Britain since 1945’, in P. Catterall and J. Obelkevich, eds, *Understanding Post-War British Society* (London, 1994), 64.

29 To calculate this midpoint, we took the maximum percentage of women securing a particular qualification in the cohort and subtracted the minimum possible percentage of women securing a particular qualification.

30 For discussion of the range of qualifications at 16 see O. Banks, *Parity and Prestige in English Secondary Education* (London, 1955), 206–09 and Mandler, *Crisis of the Meritocracy*, 26–42 and 83–84. As explained in footnote 29, the available data does not allow for differentiation between these forms of qualification. This necessarily collapses distinctions that would have been of significance to contemporaries. For the mothers’ and subjects’ generation, scaling measures have been used which do allow for differentiation between school-based and vocational qualifications at 16 and 16+ and between those securing ‘Basic’ GCE O-Level/GCSE qualifications or equivalent and those with 5+ O-Level/GCSE qualifications or equivalent.

31 E. Worth, ‘Women, Education and Social Mobility in Britain during the Long 1970s’, *Cultural and Social History*, 16 (2019), 69. N. Thomlinson, ‘Education, Class, Gender and Social Mobility in Post-war Britain’s Coalfield Communities’ (SESC Blog, 8 March 2019), <http://sesc.hist.cam.ac.uk/2019/03/08/education-class-gender-and-social-mobility-in-post-war-britains-coalfield-communities-or-why-working-class-girls-mostly-got-working-class-jobs-but-not-always/>. (last accessed 24.11.2021)

32 S. Todd, ‘Poverty and Aspiration: Young Women’s Entry to Employment in Inter-war England’, *Twentieth Century British History*, 15 (2004), 126.

33 Todd, ‘Poverty and Aspiration’, 133.

34 D. Smith-Wilson, ‘A New Look at the Affluent Worker: The Good Working Mother in Post-war Britain’, *Twentieth Century British History*, 17 (2006), 209–10. Worth, ‘Women, Education and Social Mobility’, 77.

35 Unfortunately, data on the subjects’ partners’ education was not collected, so it has not been possible to calculate an ECC for partners in the sample.

36 Mandler, *Crisis of the Meritocracy*, chapter 3.

37 Worth, ‘Women, Education and Social Mobility’, 69. Thomlinson, ‘Education, Class, Gender and Social Mobility’.

38 L. Carter, ‘Viv and Daph’s Educational Roads to Nursing: Reflections on the History of Secondary Education and the History of Secondary Education’ <https://sesc.hist.cam.ac.uk/2018/10/02/viv-and-daphs-educational-roads-to-nursing-reflections-on-the-history-of-secondary-education-and-the-history-of-the-nhs/> (SESC Blog, 2 October 2018), 2–4 (last accessed 24.11.21).

39 The paternal cohort of 1937–41 was determined by calculating the median age of fathers in the Biobank sample.

40 L. Carter, ‘Gender’, SESC Briefing Paper, June 2018, <https://sesc.hist.cam.ac.uk/wp-content/uploads/2018/06/Briefing-paper-Gender.pdf> (last accessed 24.11.21), 6.

41 Dyhouse, ‘Graduate Mothers’, 326; McCarthy, ‘Social Science’, 291.

42 G. Jones, ‘Marriage Partners and their Class Trajectories’, in Payne and Abbott, eds, *Social Mobility of Women*, 102–9. Thompson, ‘Women, Men’, 36.

43 Dyhouse, ‘Graduate Mothers’, 326; McCarthy, ‘Social Science’, p. 292; Smith-Wilson, ‘A New Look’, 218.

44 Lawler, ‘Getting Out’, 7.

45 Dyhouse, ‘Family Patterns’, 839.

46 Thompson, ‘Women, Men’, 53.

47 Bukodi, ‘Education, First Occupation’.

